# The Role of Inhibitory Receptors in Monosodium Urate Crystal-Induced Inflammation

**DOI:** 10.3389/fimmu.2018.01883

**Published:** 2018-08-20

**Authors:** Maria J. Fernandes, Paul H. Naccache

**Affiliations:** Department of Microbiology-Infectious Diseases and Immunology, Faculty of Medicine, Laval University, CHU de Québec Research Center, Québec, QC, Canada

**Keywords:** monosodium urate crystals, inflammation, gout, inhibitory receptors, neutrophils

## Abstract

Inhibitory receptors are key regulators of immune responses. Aberrant inhibitory receptor function can either lead to an exacerbated or defective immune response. Several regulatory mechanisms involved in the inflammatory reaction induced by monosodium urate crystals (MSU) during acute gout have been identified. One of these mechanisms involves inhibitory receptors. The engagement of the inhibitory receptors Clec12A and SIRL-1 has opposing effects on the responses of neutrophils to MSU. We review the general concepts of inhibitory receptor biology and apply them to understand and compare the modulation of MSU-induced inflammation by Clec12A and SIRL-1. We also discuss gaps in our knowledge of the contribution of inhibitory receptors to the pathogenesis of gout and propose future avenues of research.

## Introduction

Crystal-induced arthropathies are a group of disorders that are triggered by crystal deposits in articular and periarticular tissues (1). Monosodium urate crystals (MSU) cause one the most common inflammatory arthritis known as gout (2). Although significant advancements have been made in our understanding of the pathogenesis of this very painful and usually self-limiting arthritis, the role of immune inhibitory receptors in gout is only starting to emerge.

**Figure 1:**
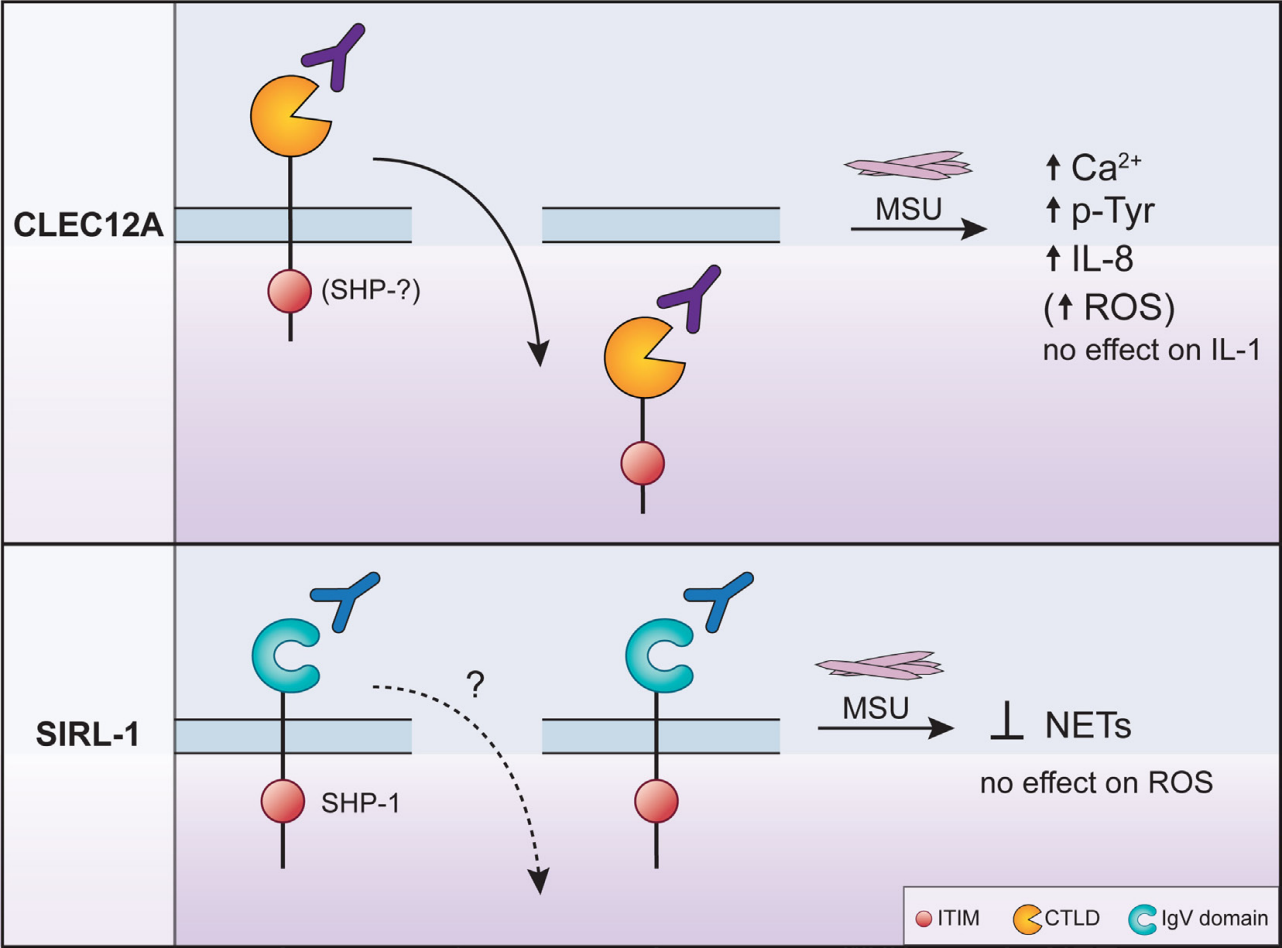
The regulation of monosodium urate crystal (MSU)-induced neutrophil activation by CLEC12A and SIRL-1 after engagement with specific antibodies. The engagement of CLEC12A on the surface of human neutrophils with a specific antibody (50C1) induces its internalization (*solid arrow pointing downwards*) resulting in the enhancement of the signaling events and effector functions shown in the figure. The phosphatases that interact with the immunoreceptor tyrosine-based inhibitory motif (ITIM) of CLEC12A in primary cells remain unknown (*top panel*). By contrast, the ligation of SIRL with the anti-SIRL-1 antibody inhibits neutrophil extracellular trap (NET) formation. In primary cells, the ITIM of SIRL-1 interacts with SHP-1. The MSU-induced responses that are not regulated by either receptor are also shown and underscore the specificity of inhibitory receptor function. The anti-SIRL-1 antibody has therapeutic potential since it inhibits NET formation.

Inhibitory receptors play key roles in regulating almost every aspect of an immune response mainly by blocking activating pathways (3–5). The integration of activating and inhibitory signals by leukocytes determines the nature of an immune response. Among the vast repertoire of inhibitory receptors expressed by neutrophils, CLEC12A and SIRL-1 modulate MSU-induced inflammation. The ligation of these two receptors has opposing effects on certain neutrophil effector functions induced by MSU offering novel insights into alternative mechanisms through which inflammation in gout can be regulated (Figure 1). Herein, we provide a brief overview of the immunopathogenesis of gout and an overview of the biology of inhibitory receptors, their role in MSU-induced inflammation, as well as their therapeutic potential and suggestions for future research.

## MSU-Induced Inflammation

Monosodium urate crystals are a crystallized form of uric acid, a product of purine metabolism (6). Uric acid is ubiquitous and only becomes inflammatory when it crystallizes to form MSU. Since MSU form in individuals with serum levels of uric acid that are chronically above the saturation point, the first line of treatment of gout is the pharmacological reduction of uric acid (7).

Monosodium urate crystals are a potent pro-inflammatory stimulus for professional phagocytes, the main drivers of acute gouty arthritis (8, 9). When these crystals accumulate in the joint, they activate resident mononuclear phagocytes to release several inflammatory mediators including IL-1β. The key role of IL-1β plays is underscored by the ability of IL-1 inhibitors to diminish the symptoms of an acute gout attack (10). IL-1β induces the expression of adhesion molecules by endothelial cells and the production of chemokines that promote a massive influx of neutrophils into the joint (11, 12). The recruited neutrophils very effectively drive the inflammatory reaction by secreting S100A9 proteins and pro-inflammatory cytokines including the potent chemoattractant for neutrophils IL-8 (13). Neutrophils also release reactive oxygen species (ROS) and degradative enzymes that cause joint destruction. The pivotal role of the neutrophil in gout is supported by a significant decrease in MSU-induced inflammation in dogs depleted of neutrophils (14, 15). Monocytes are also recruited to the affected joint and contribute to gout by producing pro-inflammatory cytokines such as IL-1 and IL-6.

Monosodium urate crystals activate a unique subset of signaling pathways in the neutrophil [reviewed in Ref. (13)]. These include the Src-family kinases (e.g., Lyn), PKC, PI-3K, Tec, and Syk. Syk is required for the MSU-induced increase in intracellular calcium levels, the production of superoxide and potentially the activation of MAP kinases. The MSU-induced synthesis of cytokines and degranulation depends on the activation of PI-3K.

One intriguing feature of acute gouty arthritis is its spontaneous resolution, typically within 7–10 days (16). Neutrophils not only secrete pro-inflammatory mediators when activated by MSU but also release neutrophil extracellular traps (NETs) (17–19). These extracellular web-like structures composed of decondensed DNA and neutrophil granule enzymes contribute to the resolution of inflammation by densely clustering MSU within them forming structures known as aggregated NETs or tophi (18, 19). Aggregated NETs form when neutrophils are present at high density which is typical of a gout attack (20). NETs also degrade pro-inflammatory cytokines and chemokines that become trapped in these structures dampening the inflammatory reaction. Additional mechanisms that terminate acute gout include the coating of MSU with lipoproteins, the production of TGFβ, IL-10, and the clearance of apoptotic neutrophils (21). IL-37, a cytokine produced by monocytes and dendritic cells, downregulates the MSU-induced production of cytokines and dampens neutrophil recruitment in mouse models of gout (22).

Negative regulatory mechanisms not only dampen a gout attack but also influence its initiation by modulating the threshold of activation of leukocytes. These negative regulatory pathways are downstream effectors of inhibitory receptors expressed on the surface of the cell. Each leukocyte expresses a diverse repertoire of inhibitory receptors (4). The challenge to understanding the role of these receptors in gout is to identify those that regulate the mechanisms underlying MSU-induced inflammation.

## How Do Inhibitory Receptors Work? General Concepts

Inhibitory receptors are essential for the maintenance of immune homeostasis as well as the termination of an immune response by blocking signaling pathways that lead to cellular activation (23). Aberrant inhibitory receptor function could either cause anergy or an excessive immune response.

Inhibitory receptors are composed of a variable number of extracellular ligand-binding domains, a transmembrane domain and a cytoplasmic tail that contains at least one immunoreceptor tyrosine-based inhibitory motif (ITIM) (3). The extracellular ligand-binding domains of inhibitory receptors come in two flavors, those that have the structure of the immunoglobulin superfamily (IgSF) domain and those with a C-type lectin-like domain (CTLD) (24).

Once engaged by a ligand, inhibitory receptors signal through their ITIM(s) (23) that is composed of six amino acids, (I/V/L/S)xYxx(L/V) (“x” denotes any amino acid, “Y” is tyrosine). Briefly, engagement of inhibitory receptors induces their clustering and the phosphorylation of the tyrosine residue of their ITIM(s) by a Src kinase that serves as a ligand for the subsequent recruitment of cytoplasmic phosphatases such as SHP-1, SHP-2, and SHIP to the plasma membrane. These phosphatases dephosphorylate signaling proteins involved in activating pathways. Inhibitory receptors can also negatively regulate cellular activation by binding adaptor proteins such as the negative regulator of the Src family, Csk (c-Src tyrosine kinase), Dok adaptor proteins (Downstream of Kinase), or Cbl (Casitas B-lineage lymphoma). These events likely occur in specialized membrane domains such as lipid rafts as has been described in lymphocytes (25).

Inhibitory receptors regulate many aspects of leukocyte development and function (3). Some of the functions they regulate in phagocytes include phagocytosis, the production of ROS and cytokines (4, 5). Although there is some degree of redundancy in their function, these receptors also differentially affect leukocyte function as illustrated by SIRL and CLEC12A in MSU-induced inflammation (discussed below). A loss of function of an inhibitory receptor can thus dysregulate multiple leukocyte functions and cause inflammatory diseases.

## The Role of Inhibitory Receptors in MSU-Induced Inflammation

To understand the role of inhibitory receptors in an inflammatory response, the modulation of their expression and their clustering, the phosphorylation of their ITIM motif(s), and the recruitment of protein partners are studied *in vitro*. Although these experimental approaches only offer us a snapshot of the involvement of these inhibitory receptors in the continuum of the cellular and chemical events of an inflammatory response, they provide insight into how these receptors work. Experiments in knock-out mice complement *in vitro* studies by revealing the outcome of the integration of activating and inhibitory signals in a specific inhibitory receptor-deficient background.

Studies in neutrophils identified two inhibitory receptors associated with MSU-induced inflammation, namely, MICL (the myeloid inhibitory C-type lectin-like receptor, CLEC12A, DCAL-2, CLL-1, and CD371) and the IgSF receptor known as SIRL-1 (the signal inhibitory receptor on leukocytes-1). The ligation of these receptors regulates MSU-induced activation of neutrophils in different ways underscoring the ability of inhibitory receptors to differentially regulate leukocyte function.

### CLEC12A

CLEC12A belongs to the C-type lectin superfamily of proteins. It harbors an extracellular CTLD linked to its transmembrane domain by a neck domain and one ITIM motif (VTYADL) in its cytoplasmic tail (26–28). CTLDs bind a diverse array of glycan ligands of endogenous or microbial origin (29–33) in a calcium-dependent manner or *via* alternative mechanisms. They can also bind non-glycan ligands (e.g., proteins and lipids). CLEC12A is devoid of the known amino acid required for glycan and calcium binding in its CTLD, rendering the identification of its natural ligands a challenge. Murine CLEC12A binds various mouse tissues, the identity of which remains to be determined (34, 35). Regarding the neck domain, it allows the oligomerization of CLRs increasing their affinity for their ligand (36, 37). It remains unknown whether human CLEC12A oligomerizes in primary cells under resting and/or stimulated conditions. Human CLEC12A is predominantly a myeloid receptor but is also expressed in some lymphocyte populations (e.g., B cells) (26–28, 38, 39). As for the underlying molecular mechanisms through which CLEC12A regulates leukocyte functions, its ITIM binds SHP-1 and SHP-2 in RAW cells (Table 1) (26). This observation requires confirmation in human primary cells.

**Table 1:** Overview of the molecular mechanisms that govern CLEC12A and SIRL-1 function in different cell types.

CLEC12A						SIRL-1					
Antibody	None	50C1	None	hDCAL-2	hDCAL-2	hDCAL-2	5D3	None	Anti-SIRL-1	Anti-SIRL-1	Anti-SIRL-1
Cell type	RAW	Human neutrophil	Clec12A KO neutrophils	iDC	iDC	iDC	MDDC	Human monocyte	RBL-2H3 (and 293 cells)	RBL-2H3	Human neutrophil
Stimulus	Pervan.	MSU	MSU	None	LPS/zymosan	CD40L-Fc	CCL2	Pervan.	Pervan.	Anti-TNP IgE	MSU (or ops. bacteria)
Receptor internalization		Yes		Yes	Yes	Yes	Yes				
Co-IP	SHP-1 and SHP-2							SHP-1	SHP-1 (SHP-2 in 293 cells)		
Signaling		↑ [Intracellular calcium], global tyrosine phospho	↑ Phospho. p40^phox^	↑ Tyrosine phospho pp38 and ppERK					ITIM phospho		
Functions enhanced		IL-8 release	ROS	CCR7 expression and production of TNFα, IL-6, IL-10, and MIP-3β	CCR7, DC-LAMP, expression	CCR7, DC-LAMP, CD83, CD86, IL-12, IL-6, IL-10, and TNFα expression; co-cultured T cell production of IFN-γ					
Functions diminished or inhibited					IL-12 (and TNFα for LPS)		Migration toward CCL2			Degranulation	NET release
Functions not affected		IL-1 production	IL-1 production		IL-6 and IL-10 production						ROS (or intra-cellular killing)

*The studies were chosen according to the insight they provide on the molecular mechanisms governing CLEC12A and SIRL-1 function. The information in this table does not cover all the published findings on these receptors. Additional references can be found in the text. The signaling events downstream of both receptors and their effect on cell function depend on the cellular context and type of stimulus*.

*iDC, immature dendritic cells; MDDC, monocyte-derived dendritic cells; Pervan., pervanadate; phospho, phosphorylation; ops, opsonized; NET, neutrophil extracellular trap; ITIM, immunoreceptor tyrosine-based inhibitory motif; ROS, reactive oxygen species; MSU, monosodium urate crystals*.

It is now widely accepted that one of the key roles of CLEC12A is to negatively regulate myeloid cell function and that it is associated with several inflammatory diseases (40–44). The function of CLEC12A is regulated by changes in its expression (26–28). TLR ligands (e.g., LPS and pam2csk4), for instance, downregulate CLEC12A expression in human monocytes. In a human skin abrasion model of inflammation, the expression of CLEC12A is also reduced (45). The effect of the downregulation of CLEC12A expression was studied in several cell types (Table 1). In monocyte-derived dendritic cells, the internalization of CLEC12A induced by its ligation with a specific antibody suppressed TLR-induced cytokine production such as IL-12 (27). By contrast, the expression of CCR7 was enhanced (27). Together, these observations indicate that CLEC12A expression is downregulated in response to various inflammatory stimuli that signal through different receptors resulting in the modulation of leukocyte activation.

Several lines of evidence indicate that CLEC12A negatively regulates myeloid cell function in MSU-induced inflammation. The stimulation of neutrophils with MSU crystals downregulates the cell-surface expression of CLEC12A (40). CLEC12A is not shed, but is internalized and degraded. When the cell-surface expression of CLEC12A is downregulated with a specific antibody, an enhancement in the MSU-induced increase of the concentration of intracellular free calcium, the tyrosine phosphorylation of proteins, and the release of IL-8 is observed. Similar observations were made in a neutrophil-like cell line in which CLEC12A expression was silenced (40). Similar to other inhibitory receptors, CLEC12A exhibits selectivity in its suppressive properties. Although it dampens the release of IL-8 induced by MSU, it does not regulate the MSU-induced secretion of IL-1 by human neutrophils. Our observations *in vitro* were corroborated *in vivo* in a knock-out mouse model of CLEC12A (41). MSU-induced inflammation in these mice was characterized by a significant increase in the recruitment of leukocytes to the site of inflammation. Of pertinence to gout, is the production of significantly higher amounts of ROS by CLEC12A deficient, mouse leukocytes partly due to the phosphorylation of the p40^phox^, a subunit of NADPH oxidase (41). It remains to be determined whether CLEC12A modulates the production of ROS in human neutrophils. Together, the above observations identify a regulatory role for CLEC12A in various leukocyte effector functions relevant to the immunopathogenesis of gout.

### SIRL-1

SIRL-1 is an IgSF receptor that is expressed on myeloid cells including neutrophils, eosinophils, monocytes, and dendritic cells (46). SIRL harbors one extracellular IgV domains and two ITIMs (VtYaeL and HeYaaL) in its cytoplasmic portion that recruit SHP-1 and SHP-2 indicative of a negative regulatory function for this receptor [Table 1; (46)].

SIRL-1 negatively regulates innate immune responses toward various stimuli. As is the case with CLEC12A, the cell-surface expression of SIRL-1 diminishes when certain stimuli activate monocytes and neutrophils (47, 48). The decrease in expression may enhance cell activation since monocytes with a low expression of SIRL-1 produce more TNFα in response to Curdlan than SIRL^high^ monocytes. While a decrease in SIRL-1 expression enhances cell activation, its ligation with a specific antibody (anti-SIRL) has the opposite effect. In the RBL-2H3 cell *in vitro* model of FcεRI activation, for instance, ligation of SIRL-1 with anti-SIRL antibody inhibited IgE-induced degranulation [Table 1; (46)]. Likewise, in human neutrophils, SIRL-1 ligation suppresses NET formation induced by opsonized *S. aureus* but not the production of ROS, thereby underscoring a certain degree of functional selectivity (49).

SIRL-1 also inhibits neutrophil activation in response to MSU. The ligation of SIRL-1 with anti-SIRL suppresses MSU-induced formation of NETs (49). This inhibitory effect on the release of NETs seems specific for stimuli that signal through Fc receptors since MSU-induced NET formation is Fcγ receptor-dependent and SIRL does not downregulate the formation of NETs by neutrophils stimulated with non-opsonized *S. aureus* or LPS (49, 50). It is noteworthy that SIRL-1 does not dampen extracellular ROS production in response to MSU which is consistent with the fact that MSU-induced formation of NETs is ROS independent. Together, these observations indicate that SIRL suppresses MSU-induced NET release in a ROS-independent but FcR-dependent manner.

## What Induces the Engagement of Inhibitory Receptors in the Context of MSU-Induced Inflammation?

The evidence that CLEC12A and SIRL-1 modulate MSU-induced neutrophil activation was obtained from experiments performed with naked crystals. MSU thus trigger activating signaling pathways by directly interacting with components of the cell surface. Since MSU are composed of a rugged, crystalline surface that is negatively charged, it is difficult to envisage how such a surface can exhibit the same level of specificity as a conventional “hand in the glove” receptor–ligand interaction. One receptor through which MSU activates signaling in human neutrophils is the Fc receptor FcγRIIIb (50). Since there are no antibodies on naked crystals, MSU seem to act as an opportunistic ligand for Fc receptors. It stands to reason that the same applies to the other receptors with which MSU have been shown to interact including CLEC12A, CD11b, CD14, TLR2, and TLR4 (6, 13, 41, 50). It is likely that MSU induces the clustering of these receptors to induce their activation, possibly in signaling hubs located in specialized membrane domains as reported for other inhibitory receptors causing cellular activation (25). An elegant study provided evidence that MSU interacts with components of cellular membranes, particularly cholesterol, supporting this notion (51). These electrostatic interactions induce changes in the composition and distribution of lipid-rich domains of the plasma membrane and most likely cause the clustering of receptors on the cell surface resulting in cell activation. Immune receptors signal through these membrane domains (25).

## What Can We Learn about the Modulation of MSU-Induced Neutrophil Activation by CLEC12A and SIRL-1?

CLEC12A and SIRL-1 elegantly illustrate the ability of inhibitory receptors to have distinct effects on leukocyte activation. Whereas the ligation of SIRL-1 with a specific antibody leads to the downregulation of cellular effector functions, the antibody-induced ligation and internalization of CLEC12A enhances cellular activation. This implies that the downregulation of the cell-surface expression of CLEC12A releases its inhibitory effect and consequently lowers the threshold of activation of neutrophils toward MSU. These observations suggest that CLEC12A may be constitutively phosphorylated in resting cells to increase the threshold of activation. The phosphorylation status of the ITIM of CLEC12A remains, however, to be determined in resting and stimulated cells. With regards to SIRL-1, it is most likely constitutively phosphorylated since it associates with SHP-1 in resting monocytes. This notion is supported by the inhibition of the MSU-induced formation of NETs after the ligation of SIRL-1. Since the anti-SIRL-1 antibody inhibits MSU-induced formation of NET, it is an agonistic antibody. By contrast, the anti-CLEC12A antibody does not seem to have agonistic effects since it enhances cell activation by binding CLEC12A. Although the role of CLE12A and SIRL-1 in gout remains to be fully characterized, these two receptors may regulate neutrophil activation in a temporal manner since NET formation is a later response to MSU than the mobilization of calcium.

## Where Should We Go from Here?

To develop a more complete picture of the immunopathogenesis of gout, the constellation of inhibitory receptors that regulate myeloid cell responses toward MSU needs to be identified. Moreover, it is essential to identify the ligands for these receptors to better understand their functional and temporal role during homeostasis and inflammation as well as their mode of action. Pyz et al. (34) reported that CLEC12A binds endogenous ligands whose identity remains to be determined. *In vivo* studies will also be informative. With regards to CLEC12A, for instance, it may also contribute to the resolution of inflammation in gout since the arthritis phenotype in CLEC12A knock-out mice persists for a significantly longer period of time than in wild-type mice (42).

## Are Inhibitory Receptors a Clinically Relevant, Therapeutic Target for Gout?

The dampening of MSU-induced neutrophil activation by the SIRL-1 antibody supports the notion that targeting myeloid inhibitory receptors is a promising and new therapeutic option for the treatment of gout. Inhibitory receptors could also be targeted with compounds that preserve their expression once the mechanisms underlying their internalization and degradation are characterized. Targeting inhibitory receptors that regulate the threshold activation of neutrophils during the early stages of a gout attack would be beneficial since there is a therapeutic window of opportunity prior to the peak of a gout attack during which patients feel a slight pain. Colchicine is one such drug that effectively reduces inflammation if administered during the early phase of an acute gout attack. A mechanism through which colchicine may dampen a gout attack is by preserving the cell-surface expression of CLEC12A on neutrophils (40).

It is difficult to predict whether targeting endogenous inhibitory pathways will cause less adverse effects than currently used drugs. Considering the limited choice of anti-inflammatory drugs to treat gout due, in part, to contraindications in many cases, it is timely to consider developing additional drugs to treat gout attacks (52). A recent drug developed to treat gout, anti-IL-1 is efficacious but further cost/benefit and safety studies are required prior to widely using it to treat this arthropathy (10).

It is an exciting time for inhibitory receptor research since we have better tools to study their biology and a clearer picture of how they work facilitating the investigation of their function and role in disease. At least two other inhibitory pathways in lymphocytes are targeted for the treatment of rheumatoid arthritis and cancer (53) providing a proof of concept of the therapeutic value of targeting inhibitory receptors to treat disease. Considering that gout is one of the few types of arthritis whose causative agent is known, defining the role of inhibitory pathways in this disease will serve as a paradigm for understanding the pathogenesis of other types of arthritis and identifying novel therapeutic targets.

## Author Contributions

MF and PHN contributed equally to the writing of the manuscript.

## Conflict of Interest Statement

The authors declare that the research was conducted in the absence of any commercial or financial relationships that could be construed as a potential conflict of interest.
